# Impact of age on sorafenib outcomes in hepatocellular carcinoma: an international cohort study

**DOI:** 10.1038/s41416-020-01116-9

**Published:** 2020-10-19

**Authors:** Saur Hajiev, Elias Allara, Leila Motedayеn Aval, Tadaaki Arizumi, Dominik Bettinger, Mario Pirisi, Lorenza Rimassa, Tiziana Pressiani, Nicola Personeni, Laura Giordano, Masatoshi Kudo, Robert Thimme, Joong-Won Park, Tamar H. Taddei, David E. Kaplan, Ramya Ramaswami, David J. Pinato, Rohini Sharma

**Affiliations:** 1 Division of Surgery and Cancer, Imperial College London, Hammersmith Hospital, Du Cane Road, London, W12 0NN UK; 2 Department of Public Health and Primary Care, University of Cambridge, Cambridge, UK; 3 Department of Gastroenterology and Hepatology, Kinki University School of Medicine, Osaka-Sayama, Japan; 4 Department of Medicine II, University Medical Center, Freiburg, Germany; 5 Berta-Ottenstein Programme, Faculty of Medicine, University of Freiburg, Freiburg, Germany; 6 Department of Translational Medicine, Università degli Studi del Piemonte Orientale, Novara, Italy; 7 Medical Oncology and Hematology Unit, Humanitas Cancer Center, Humanitas Clinical and Research Center-IRCCS, Rozzano (Milan), Italy; 8 Department of Biomedical Sciences, Humanitas University, Pieve Emanuele (Milan), Italy; 9 National Cancer Centre Hospital, Goyang, South Korea; 10 University of Pennsylvania Perelman School of Medicine, Philadelphia, PA USA

**Keywords:** Targeted therapies, Hepatocellular carcinoma

## Abstract

**Background:**

There is no consensus on the effect of sorafenib dosing on efficacy and toxicity in elderly patients with hepatocellular carcinoma (HCC). Older patients are often empirically started on low-dose therapy with the aim to avoid toxicities while maximising clinical efficacy. We aimed to verify whether age impacts on overall survival (OS) and whether a reduced starting dose impacts on OS or toxicity experienced by the elderly.

**Methods:**

In an international, multicentre cohort study, outcomes for those aged <75 or ≥75 years were determined while accounting for common prognostic factors and demographic characteristics in univariable and multivariable models.

**Results:**

Five thousand five hundred and ninety-eight patients were recruited; 792 (14.1%) were aged ≥75 years. The elderly were more likely to have larger tumours (>7 cm) (39 vs 33%, *p* < 0.01) with preserved liver function (67 vs 57.7%) (*p* < 0.01). No difference in the median OS of those aged ≥75 years and <75 was noted (7.3 months vs 7.2 months; HR 1.00 (95% CI 0.93–1.08), *p* = 0.97). There was no relationship between starting dose of sorafenib 800 mg vs 400 mg/200 mg and OS between those <75 and ≥75 years. The elderly experienced a similar overall incidence of grade 2–4 sorafenib-related toxicity compared to <75 years (63.5 vs 56.7%, *p* = 0.11). However, the elderly were more likely to discontinue sorafenib due to toxicity (27.0 vs 21.6%, *p* < 0.01). This did not vary between different starting doses of sorafenib.

**Conclusions:**

Clinical outcomes in the elderly is equivalent to patients aged <75 years, independent of dose of sorafenib prescribed.

## Background

Hepatocellular carcinoma (HCC) is a leading cause of cancer death with >850,000 cases diagnosed yearly worldwide with a similar annual mortality.^1^ If HCC presents at an early stage, curative options such as liver transplantation, resection or percutaneous radiofrequency ablation are feasible; however, this is only possible in 30–40% of patients with the majority undergoing non-curative treatments such as transcatheter arterial chemoembolisation or systemic treatment.^2^

Sorafenib is a multi-kinase inhibitor that interferes with intracellular and extracellular signalling pathways associated with tumour angiogenesis and tumour proliferation, including vascular endothelial growth factor receptors, platelet-derived growth factor receptor and RAF/mitogen-activated protein kinase.^3^ The SHARP and Asia-Pacific studies confirmed overall survival (OS) benefit of sorafenib over placebo; however, the median age of patients in both studies was <65 years.^4,5^

Age is a risk factor for developing HCC with the highest age-specific incidence in most Western populations occurring over 75 years, a pattern that is replicated in Asia.^6–8^ The number of elderly patients with HCC is likely to increase not only as a result of demographic trends but also due to improvements in treatment of chronic liver disease. Comorbidities and frailty in the elderly often makes them unsuitable for surgical and locoregional treatment—placing even more importance on clear guidelines for medical therapy. The safety of sorafenib treatment in elderly patients is of particular concern due to comorbidities, impaired organ function, polypharmacy as well as altered pharmacokinetics; sorafenib is metabolised by CYP3A, the activity of which slows with increasing age.^9^ There is a paucity of evidence in terms of safety, efficacy and recommended dosing for the elderly due to their under-representation in clinical trials. It has been suggested that the clinical outcome from sorafenib is not influenced by increasing age.^9–13^ In a multicentre study in Japan, 179 patients aged ≥75 years receiving sorafenib were matched to 279 patients aged <75 years.^14^ No difference in efficacy or tolerability was reported. Conversely, Edeline et al. and Williet et al. reported a greater discontinuation rate due to toxicity among older populations and greater incidence of serious adverse events (AEs) in the latter.^15,16^ Real-world studies illustrate that patients aged >70 years are more likely to be started on 200/400 mg daily as opposed to standard dosing with 800 mg/daily.^14,17,18^ Two studies found that sorafenib was better tolerated in the elderly when physicians commenced on a reduced initial dose but there remains a lack of consensus on initial dose of sorafenib among elderly patients.^19,20^ To our knowledge, previous studies evaluating treatment of HCC with sorafenib in an elderly population have been based on a single geographical region. There is a clear need for international data as the differing aetiology of HCC globally can impact on outcomes of treatment.^12^

To address this issue and to overcome the limitations of previous studies, we designed a large collaborative global study with the primary objective to assess the effect of age on OS in patients treated with sorafenib while accounting for common prognostic factors. As a secondary endpoint, we investigated the impact of the starting dose of sorafenib in the elderly population aged ≥75 years on OS and drug tolerability.

## Methods

Five thousand five hundred and ninety-eight consecutive patients who had undergone treatment with sorafenib from 2007 to 2018 were recruited from seven centres: Veterans Health Administration (VHA) Hospitals, USA (4903 patients, 87.6%); Kinki University School of Medicine, Japan (194 patients, 3.5%); Humanitas Clinical and Research Centre, Milan, Italy (168 patients, 3.0%); Imperial College NHS Healthcare Trust, UK (113 patients, 2%); University Medical Centre Freiburg, Germany (71 patients, 1.3%); National Cancer Centre Hospital, South Korea (97 patients, 1.7%); and Università degli Studi del Piemonte Orientale, Novara, Italy (52 patients, 0.9%). We accessed prospectively collected cohorts of patients who had a diagnosis of HCC, except from the VHA hospitals, where data were retrospectively collected. Consecutive patient data were collected as part of routine clinical care. For prospective collection, patients were either Child–Turcotte–Pugh (CTP) grade A liver impairment or CTP B with low disease burden. The VHA cohort included patients of all CTP liver classes. All patients were discussed at a multidisciplinary team meeting, where they were deemed not suitable for curative or locoregional treatment. No patients had previous systemic therapy. Patients were either started on 200 or 400 mg once daily or 400 mg twice daily (800 mg/day), depending on clinical assessment. Treatment with sorafenib continued until significant toxicity from treatment, withdrawal of consent or disease progression.

Toxicity related to sorafenib, including hand–foot skin reaction (HFSR), diarrhoea, liver dysfunction, mucositis, rash, constipation, anorexia, fatigue and hypertension, was evaluated using the National Cancer Institute Common Terminology Criteria for Adverse Events 4.03. Information on whether further dose reductions were required was obtained. OS was taken from the date of sorafenib commencement to the date of progression, death on treatment or last follow-up. The study protocol was approved by the institutional review board or ethics committee in each participating institution and was conducted in accordance with the Declaration of Helsinki (update 2004).

### Statistical analysis

The primary endpoint was OS comparing patients aged <75 and ≥75 years. Secondary outcomes were the incidence of AEs and the impact of dose on incidence of AEs and OS. Continuous variables were expressed as median. Categorical variables were expressed as frequencies with percentages. Variables were compared between younger (<75 years) and the elderly (≥75 years) using Mann–Whitney *U* test for continuous variables and *χ*^2^ test or Fisher’s exact test for categorical variables, as appropriate. Other prognostic variables considered were BCLC stage (AB vs CD), AFP (≤ or >400 ng/dL), tumour size (≤ or >7 cm), aetiology of liver disease, presence of portal vein thrombosis (PVT), sorafenib starting dose (800 mg vs 200/400 mg), presence of metastases and country of origin. The survival function by age and potential prognostic factors was plotted using Kaplan–Meier curves with differences between these groups compared using log-rank test. Before conducting Cox regression analyses, the proportional hazards assumption was checked with log-likelihood ratio tests of each predictor over time bands. There was no evidence of violation of the proportional hazard assumption for the main predictor (age; *p* = 0.81). For the predictors for which the proportional hazards assumption did not hold, time-dependent effects were explicitly accounted for by adding interaction terms with time bands in all regression analyses.^21^

Due to the considerable proportion of missing data for some variables (up to ~90% for the full data set of 5598 patients and up to ~70% for the subset of 792 patients with age ≥75 years), a multiple imputation with chained equations (MICE) approach was used, assuming that the data were missing at random. Each data set was imputed separately to allow for imputation of prognostic factor specific to elderly patients. For both the full data set of 5598 patients and the subset of 792 elderly patients, we included in the imputation models (i) all predictors of missingness (log-likelihood *p* < 0.10) for any of the variables included in the initial analysis model specified above, (ii) the event indicator (subject alive/death), and (iii) the Nelson–Aalen estimator of baseline hazard.^22^ From each original data set, we generated 100 imputed data sets to provide stable estimates. Diagnostic plots of trace lines revealed satisfactory convergence of imputation models. Plots of imputed values vs the observed values suggested that imputed values were plausible.

All *p* values were derived from two-tailed tests, and *p* < 0.05 was defined as statistically significant. All statistical analysis was conducted using SPSS statistical package version 26 (SPSS Inc., Chicago, IL, USA) and R version 3.61 (Copyright 2019 The R Foundation for Statistical Computing) with packages *mice* 3.7.0 and *survival* 3.1–8.

## Results

### Baseline characteristics of the study population

Five thousand five hundred and ninety-eight patients were included. Of these, 792 (14.1%) were equal to or over the age of 75 years (Table 1). The mean age of the study population was 64.3 years. The seven centres had different distributions of ages, with the Korean centre almost exclusively treating patients aged <75 years (97.9%). The majority of the study population were men (96.5%) and had CTP A stage liver cirrhosis (58.9%). Hepatitis C virus (HCV) infection and alcohol misuse were the primary underlying causes of liver disease (51.7 and 46.0%, respectively), although several patients had multiple aetiological factors.

**Table 1:** Baseline characteristics of the study population.

Baseline characteristic	All patients (%), *N* = 5598	Age <75 (%), *N* = 4806 (85.8)	Age ≥75 (%), *N* = 792 (14.1)	*p* value
Centre				<0.01
USA	4903 (87.6)	4318 (89.8)	585 (73.8)	
Japan	194 (3.5)	108 (2.2)	86 (10.9)	
Milan, Italy	168 (3.0)	120 (2.5)	48 (6.1)	
UK	113 (2.0)	81 (1.7)	32 (4.0)	
South Korea	97 (1.7)	95 (2.0)	2 (0.3)	
Germany	71 (1.3)	53 (1.1)	18 (2.3)	
Novara, Italy	52 (0.9)	31 (0.6)	21 (2.6)	
Sex				<0.01
Male	5402 (96.5)	4667 (97.1)	735 (92.7)	
Female	196 (3.5)	139 (2.9)	57 (7.2)	
Risk factors for chronic liver disease^a^				
Hepatitis B infection	395 (7.1)	366 (7.6)	29 (3.7)	<0.01
Alcohol related^b^	2368 (43.8)	2244 (47.8)	124 (17.6)	<0.01
Hepatitis C infection	2896 (51.7)	2722 (56.6)	174 (22.0)	<0.01
Other^c^	237 (4.3)	156 (3.3)	81 (10.3)	<0.01
Child–Turcotte–Pugh class				<0.01
A	3281 (58.9)	2754 (57.5)	527 (67.0)	
B	2071 (37.2)	1821 (38.0)	250 (31.8)	
C	220 (3.9)	211 (4.4)	9 (1.1)	
Maximum tumour diameter				<0.01
≤7 cm	2236 (66.1)	1914 (67.0)	322 (60.9)	
>7 cm	1148 (33.9)	942 (33.0)	206 (39.0)	
Portal vein thrombus				<0.01
Absent	3633 (71.6)	3070 (70.6)	563 (77.9)	
Present	1441 (28.4)	1281 (29.4)	161 (22.1)	
AFP (ng/dL)				0.05
≤400	2523 (47.9)	2160 (47.4)	363 (51.5)	
>400	2743 (52.1)	2401 (52.6)	342 (48.5)	
Metastases				0.3
Absent	4381 (79.0)	3778 (79.2)	603 (77.5)	
Present	1168 (21.0)	993 (20.8)	175 (22.5)	
Previous treatment				
Resection^d^	125 (18.0)	98 (20.1)	27 (13.0)	0.03
Radiofrequency ablation	394 (7.0)	319 (81.0)	75 (19.0)	<0.01
Transarterial chemoembolisation	792 (14.1)	231 (14.6)	561 (14.0)	0.55
Y90	5 (0.1)	5 (0.1)	0 (0.0)	0.41
Starting dose of sorafenib				0.40
200/400 mg	2095 (37.4)	1788 (37.2)	307 (38.8)	
800 mg	3503 (62.6)	3018 (62.8)	485 (61.2)	

*AFP* α-fetoprotein.

^a^Some patients often had overlapping aetiological risk factors.

^b^Alcohol data missing for Japanese patients (*N* = 194).

^c^Other aetiology data missing for Korean patients (*N* = 97).

^d^Resection data missing for US patients (*N* = 4903).

Three thousand five hundred and three patients (62.6%) were started on sorafenib 800 mg/day and 2083 (37.2%) received a reduced dose of 400 mg/day, with a median follow-up time of 15.2 months. Twelve patients (0.2%) received 200 mg/day. The proportion of patients in the two dosing groups varied significantly by centre (*p* > 0.001). Patients from Milan, Korea, USA and Japan were mostly on standard dose sorafenib (100, 89.7, 63.1 and 54.1%, respectively), whereas those in the UK, Germany and Novara were largely on reduced dose (82.3, 77.5 and 75.0%, respectively). The median duration of sorafenib treatment was 5.0 months (range 0.03–83.6 months). Progressive disease was the main cause of sorafenib cessation (56.0%) followed by toxicity (20.6%). Overall sorafenib was well tolerated; the most common severe (grade ≥2) AEs experienced were fatigue and diarrhoea, occurring in 15.2 and 17.3% of all patients, respectively.

### Age is not a significant prognostic factor with sorafenib

**Fig. 1:**
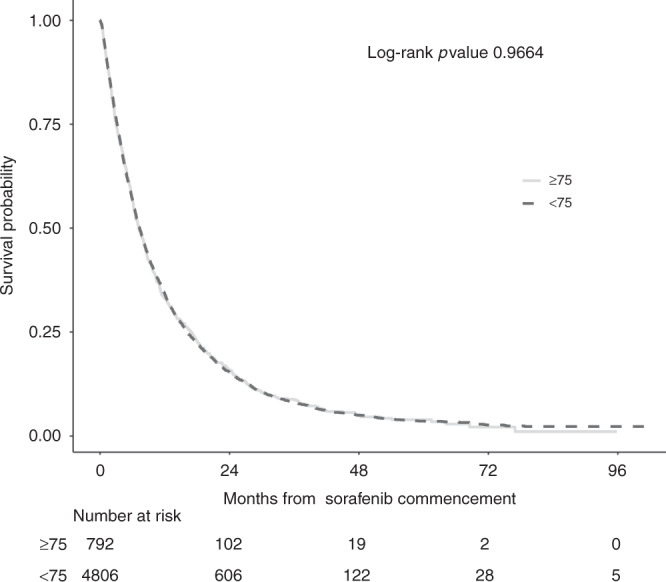
Kaplan–Meier estimates of overall survival according to age. The curves illustrate the prognostic relationship of age (<75 and ≥75 years) with overall survival in patients receiving sorafenib for HCC.

Five thousand and fifty-four patients (90.3%) died during follow-up and median OS for the cohort was 7.3 months (95% confidence interval (CI): 7.0–7.6). OS in patients aged ≥75 years did not deviate significantly from those aged <75 years (7.3 months, 95% CI: 7.0–7.6 vs 7.2 months, 95% CI: 6.4–8.0, *p* = 0.95) (Fig. 1). OS did differ significantly across the treatment centres with the longest median OS reported in Japanese, 14.3 months (95% CI: 9.2–19.4) and the shortest OS in South Korea, 5.8 months (95% CI: 4.4–7.2) (*p* = 0.01).

After adjustment for common prognostic factors and multiple imputation of missing data, the effect of age on survival remained negligible (hazard ratio (HR), 1.03 (95% CI: 0.95–1.12), *p* = 0.5). Similarly, starting dose had no impact on OS (HR 0.96, 95% CI 0.91–1.02, *p* = 0.3). Prognostic factors independently associated with OS were BCLC stage, CTP class, AFP, tumour size, presence of extrahepatic disease and geographical origin (Table 2). As expected, the adjusted effects were similar in size and direction to the complete case analysis (Supplementary Table 1), with multiple imputation analyses achieving greater precision.

**Table 2:** Effects of age and common prognostic factors on overall survival in multiple imputed data sets.

Predictor	Univariable models		Multivariable models	
	Hazard ratio (95% CI)	*p*	Hazard ratio (95% CI)	*p*
Full sample set of patients with HCC (*n* = 5598)				
Age ≥75 years	1.00 (0.93–1.08)	0.9	1.03 (0.95–1.12)	0.5
BCLC stage C or D vs 0, A or B	2.04 (1.88–2.22)	<0.001	1.57 (1.43–1.73)	<0.001
CTP class (B, C vs A)	2.28 (2.13–2.44)	<0.001	2.15 (2.01–2.30)	<0.001
Tumour size >7 cm	1.80 (1.63–1.98)	<0.001	1.57 (1.43–1.73)	<0.001
PVT	1.05 (0.98–1.12)	0.1	1.02 (0.96–1.09)	0.5
Presence of metastasis	1.50 (1.37–1.64)	<0.001	1.20 (1.08–1.32)	<0.001
AFP > 400 ng/dL	1.88 (1.74–2.04)	<0.001	1.70 (1.57–1.85)	<0.001
HCV vs other aetiologies	0.86 (0.82–0.91)	<0.001	0.88 (0.83–0.94)	<0.001
Starting dose (800 mg vs 200/400 mg)	0.94 (0.89–1.00)	0.05	0.96 (0.91–1.02)	0.29
Continent (Asia vs USA/Europe)	0.74 (0.64–0.85)	<0.001	0.78 (0.67–0.89)	<0.001
Patients aged >75 years with HCC (*n* = 792)				
BCLC stage C or D vs 0, A or B	1.50 (1.28–1.76)	<0.001	1.19 (0.98–1.45)	0.08
CTP class (B, C vs A)	2.35 (1.91–2.89)	<0.001	2.23 (1.81–2.76)	<0.001
Tumour size >7 cm	1.56 (1.30–1.87)	<0.001	1.37 (1.13–1.65)	<0.01
PVT	1.20 (1.00–1.44)	0.05	1.08 (0.90–1.30)	0.4
Presence of metastasis	1.39 (1.16–1.65)	<0.01	1.13 (0.91–1.39)	0.3
AFP > 400 ng/dL	1.70 (1.37–2.10)	<0.001	1.56 (1.26–1.95)	<0.001
HCV vs other aetiologies	0.65 (0.54–0.79)	<0.001	0.70 (0.58–0.86)	<0.001
Starting dose (800 mg vs 200/400 mg)	1.07 (0.92–1.25)	0.3823	0.95 (0.82–1.12)	0.5671
Continent (Asia vs USA/Europe)	0.60 (0.45–0.79)	<0.001	0.80 (0.60–1.07)	0.1334

*BCLC* Barcelona Cancer Liver Clinic, *CTP* Child–Turcotte–Pugh, *PVT* portal vein thrombosis, *AFP* α-fetoprotein, *HCV* hepatitis C virus.

### Prognostic factors of OS in the elderly

**Fig. 2:**
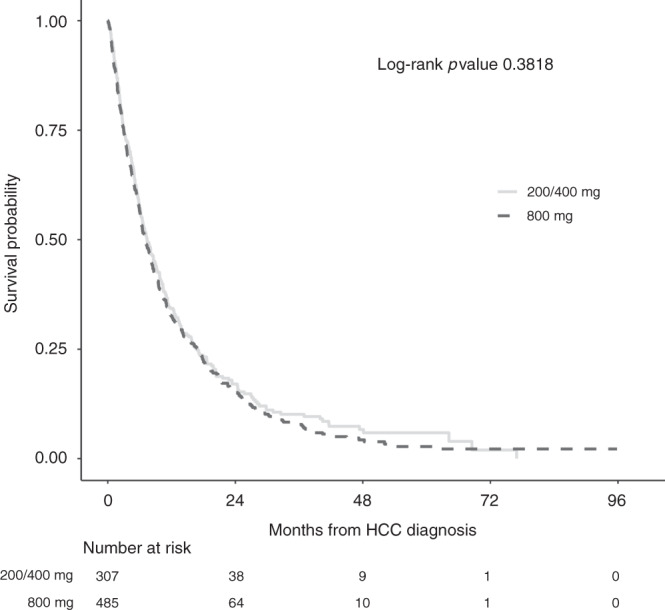
Kaplan–Meier estimates of overall survival according to starting dose of sorafenib received. Curves illustrate the prognostic relationship of starting dose (800 mg vs reduced dose) with overall survival in patients aged ≥75 years receiving sorafenib for HCC.

Of the patients aged ≥75 years, the mean age was 79.8 years (range: 75.0–93.0). Elderly patients were less likely to have HCV and hepatitis B virus (HBV) or have consumed excess alcohol (*p* < 0.001, Table 1). They also had lower AFP at diagnosis (*p* = 0.049) and less PVT compared to those aged <75 years (22.1 vs 29.4%, *p* < 0.001) but were more likely to have larger tumours (39.0 vs 33.0%, *p* = 0.007). There were fewer elderly patients with advanced CTP B and CTP C cirrhosis compared to the younger population (31.8 vs 38.0% and 1.1 vs 4.4%, respectively, *p* < 0.01). The starting dose of sorafenib did not have an impact on OS in the elderly; the median OS for the full-dose sorafenib group was 7.4 months (95% CI: 6.0–8.8) and 7.1 months (95% CI: 6.2–7.9 months) with a reduced starting dose (*p* = 0.40), (Fig. 2). Other independent prognostic identified were CTP class, AFP, tumour size and underlying disease aetiology (Table 2).

### Sorafenib-related AEs in the elderly

The incidence of grade 2–4 toxicities was similar between both age groups (63.5 vs 56.7%, *p* = 0.11, Table 3). Regarding specific AEs, the older age group had a significantly greater incidence of anorexia (14.0 vs 7.2%, *p* < 0.01) and grade ≥2 rash (6.3 vs 3.1%, *p* = 0.048) compared with the younger group. When considering the relationship between the different starting doses and incidence of toxicities in the elderly population, no difference in the incidence grade ≥2 toxicities was observed in either 800 mg or reduced dose sorafenib groups (*p* = 0.13).

**Table 3:** Incidence of adverse events (NCT CTCAE v 4.0 grade 0–1 and grade 2–4) between age groups.

Adverse event	AEs in <75 years, *n* (%)		AEs in ≥75 years, *n* (%)		*p* value
	Grade <2	Grade ≥2	Grade <2	Grade ≥2	
HFSR	424 (87.4)	61 (12.6)	175 (84.5)	32 (15.5)	0.31
Rash	473 (96.9)	15 (3.1)	194 (93.7)	13 (6.3)	0.048
Mucositis	474 (97.1)	14 (2.9)	200 (96.6)	7 (3.4)	0.72
Hypertension	451 (92.4)	37 (7.6)	183 (88.4)	24 (11.6)	0.17
Anorexia	453 (92.8)	35 (7.2)	178 (86.0)	29 (14.0)	<0.01
Fatigue	405 (83.0)	83 (17.0)	170 (82.1)	37 (17.9)	0.78
Diarrhoea	415 (85.0)	73 (15.0)	173 (83.6)	34 (16.3)	0.62
Constipation	484 (99.2)	4 (0.8)	204 (98.6)	3 (1.4)	0.45
Liver dysfunction	425 (93.2)	31 (6.8)	184 (94.8)	10 (5.2)	0.43
Other	304 (79.2)	80 (20.8)	105 (82.0)	23 (18.0)	0.48
Any adverse event grade ≥2	247 (56.7)		120 (63.5)		0.111

*HFSR* hand–foot skin reaction.

**Fig. 3:**
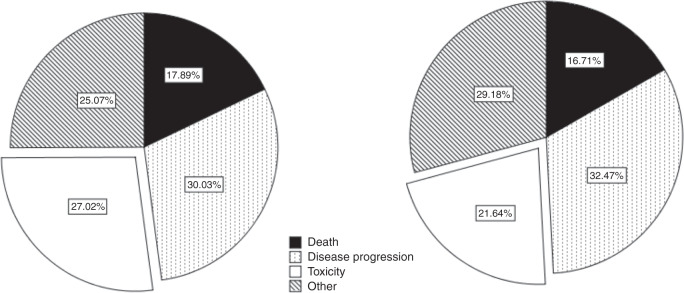
Pie charts demonstrating the reasons for sorafenib discontinuation. Reasons for discontinuation are demonstrated in patients aged ≥75 years (left) and aged <75 years (right) according to death, disease progression, toxicity or other.

More elderly patients discontinued treatment as a result of toxicity, compared to the younger patients (27.0 vs 21.6%, *p* = 0.001, Fig. 3) and this did not vary between different starting doses of sorafenib. The mean duration of treatment was similar between those aged over and under 75 years (*p* = 0.071), and again, the starting dose of sorafenib did not affect treatment duration in the elderly (*p* = 0.25). There were no differences between the age groups in the proportion of early treatment cessation at 28 days (*p* = 0.52).

## Discussion

The peak incidence of HCC occurs over the age of 75 years and yet this population group is underrepresented in clinical trials resulting in uncertainty regarding the safety and efficacy of sorafenib. As a result, clinicians extrapolate data from younger patients to the older patient group often leading to empiric dosing.^4,5,9,23^ There is a tendency to commence elderly patients on a lower starting dose of sorafenib with the aim to obviate toxicity in order to maximise duration of therapy and survival benefit. However, there is conflicting evidence for this practice and no consensus on the recommended starting dose.^10,14,16,19,20,24^ We have conducted the largest international study investigating the impact of sorafenib dosing in an elderly population, comparing clinical outcomes to patients aged <75 years.

We have conclusively shown that median OS in the elderly is the same as that in a younger patient population, regardless of dose administered. This in line with a number of smaller studies carried out in Europe and Asia; the strength of this study being that we have considered patients from Asia, Europe and the USA ensuring global representation of aetiologies of HCC and differing prescribing practices.^10,14,15,19,25,26^ While combination therapy is likely to be the new gold standard for the management of HCC, given the relative cost of sorafenib, it is unlikely that many health systems will adopt this immediately and instead will continue to utilise sorafenib.

Our study emphasises the importance of tumour-related factors and hepatic reserve in influencing survival in both the entire population studied and in those ≥75 years confirming that CTP class, tumour size and serum AFP are independent prognostic factors. Of note, the younger population was more likely to have HBV-related HCC, a more aggressive phenotype.^27,28^ Interestingly, there are clear differences in international prescribing practices in that no patient from Korea were treated with low-dose sorafenib, differences which we have highlighted in our previous studies.^29^ Of importance, the starting dose of sorafenib was not an independent predictor of OS confirming the results of the previously published work by Kaplan and colleagues, which is further strengthened in this paper by the inclusion of patients from other global regions.^30^ We also report similar frequency and severity of toxicity between the two age groups, which again reaffirms the data previously published and is in line with the incidence of toxicities reported in the SHARP and Asia-Pacific trials.^10^ The only differences of note were anorexia and rash, which was more pronounced in the elderly cohort, a key concern in maintaining quality of life in the elderly.

There is a complicated relationship between sorafenib dosing and toxicity, where toxicity is not only associated with improved outcome but also necessitates dose reductions such that, even in those patients requiring drug cessation from toxicity, the survival benefit continues beyond the drug administration period.^13,29,31^ Although this was not an endpoint of the study, we confirmed the findings of Iavarone and colleagues, in that discontinuation due to toxicity, even in the elderly, was associated with improved OS (Supplementary Fig. 1). The explanation for this remains to be ascertained with some groups proposing immune-modulating effects of sorafenib, resulting in T cell activation and infiltration of the tumour.^32^ A limitation of this study was that only starting dose was reported and not subsequent dose reductions or cumulative dose, which has been shown to impact on both duration of therapy and survival.^13^ A recent study by Tovoli and colleagues illustrates that tailoring dosing to the individual patient according to AEs experienced results in a longer duration of treatment, higher cumulative dose and an improved OS.^33^ We observed a higher rate of discontinuation of sorafenib due to toxicity in the elderly patients despite no difference in the overall incidence of AEs reported. This is mirrored by an analysis of the Celestial study by age, which confirms higher discontinuation rates in the elderly, again with no differences in toxicity.^34^ This may reflect physicians’ attitudes whereby they may be more inclined to stop treatment in the elderly even when faced with a similar safety profile as in a younger population.^35^

Chronologic age alone does not provide sufficient information on an individual’s ability to tolerate anticancer therapy, and in patients of the same age, there is wide heterogeneity in their ability to undergo therapy as a result of comorbidities, concomitant medications, altered physiologic reserve and social support. The National Comprehensive Cancer Network and the International Society of Geriatric Oncology recommend the implementation of comprehensive geriatric assessments in clinical practice to provide detailed evaluation of the health status of an older adult. The use of these tools has been shown to highlight areas of vulnerability, reduce toxicity, improve quality of life and improve prognostication in the elderly.^36^ Importantly, the use of geriatric assessments assist in treatment decision-making such that studies investigating their use illustrate treatment optimisation in up to 50% of elderly patients.^37^

The lack of clinical trial data in the elderly has long been recognised but only recently has been addressed by funding bodies such that the Food and Drug Administration and National Institutes of Health have introduced guidelines regarding trial inclusion criteria and there is a move to restrict funding for grants that have an upper age limit.^38,39^ These developments will enhance our knowledge of therapeutic efficacy of medications in the elderly.

Our study was broad in its criteria for enrolment and is thus a fair reflection of global clinical practice. Patients of various aetiologies, ages and stages of liver disease were included. The median OS in our study is lower compared to the SHARP and Asia-Pacific publications.^4,5^ This can be attributed to the inclusion of a significant number of patients with CTP B and C liver dysfunction (39%), and a significant difference was observed in the median OS between each CTP class (median OS CTPA/B/C—10.1/4.7/1.7months). The overall median OS presented in our study is in line with a meta-analysis by McNamara and colleagues who investigated the benefit of sorafenib across CTP status and reported a similar OS of 7.2 months.^40^ Patients were enrolled consecutively in all centres as to reduce selection bias. Since all centres were tertiary referral centres, the study benefitted from physician expertise. Nonetheless, there are several limitations to our study, and selection bias must be considered when evaluating our results particularly given the differences in prescribing practice across centres. Other limitations include no quantification of overall drug dose administered and the impact of dose reductions on outcome and the retrospective nature of some aspects of the data collection particularly AE reporting. The issue of missing data was addressed using multiple imputation, an approach that relies on the assumption that the data are missing at random; that is, that the missing data mechanism does not depend on unobserved data. Given the large samples size and the amount of information available in the data set, this allowed us to include in the imputation models all variables associated with missing data, rendering the missing-at-random assumption likely to hold.

## Conclusion

This large international cohort study shows that patients aged ≥75 years with HCC have an equivalent OS benefit with sorafenib to younger patients, regardless of the starting dose. We show that the overall toxicity rates in the elderly are equivalent to those aged <75 years, and the incidence and grade of toxicity experienced was independent of treatment dose received. This study highlights the importance of considering sorafenib treatment in the elderly on an individual basis as a viable treatment option.

## Supplementary information

Supplementary Materials

## Data Availability

Data are available from individual institutions.
