# Correction: Impact of age on sorafenib outcomes in hepatocellular carcinoma: an international cohort study

**DOI:** 10.1038/s41416-020-01241-5

**Published:** 2021-02-03

**Authors:** Saur Hajiev, Elias Allara, Leila Motedayеn Aval, Tadaaki Arizumi, Dominik Bettinger, Mario Pirisi, Lorenza Rimassa, Tiziana Pressiani, Nicola Personeni, Laura Giordano, Masatoshi Kudo, Robert Thimme, Joong-Won Park, Tamar H. Taddei, David E. Kaplan, Ramya Ramaswami, David J. Pinato, Rohini Sharma

**Affiliations:** 1 Division of Surgery and Cancer, Imperial College London, Hammersmith Hospital, Du Cane Road, London, W12 0NN UK; 2 Department of Public Health and Primary Care, University of Cambridge, Cambridge, UK; 3 Department of Gastroenterology and Hepatology, Kinki University School of Medicine, Osaka-Sayama, Japan; 4 Department of Medicine II, University Medical Center, Freiburg, Germany; 5 Berta-Ottenstein Programme, Faculty of Medicine, University of Freiburg, Freiburg, Germany; 6 Department of Translational Medicine, Università degli Studi del Piemonte Orientale, Novara, Italy; 7 Medical Oncology and Hematology Unit, Humanitas Cancer Center, Humanitas Clinical and Research Center-IRCCS, Rozzano (Milan), Italy; 8 Department of Biomedical Sciences, Humanitas University, Pieve Emanuele (Milan), Italy; 9 National Cancer Centre Hospital, Goyang, South Korea; 10 University of Pennsylvania Perelman School of Medicine, Philadelphia, PA USA

**Keywords:** Targeted therapies, Hepatocellular carcinoma

Correction to: *British Journal of Cancer* (2020); 10.1038/s41416-020-01116-9, published online 19 October 2020

Since the publication of this paper, the authors have noticed an error in the name of Leila Motedayеn Aval, where Miss Motedayеn Aval’s name was incorrectly listed as Leila Motedayеn-Aval. This has been corrected above.

The original article has been corrected.

