# Supplementary material for: Impact of age on sorafenib outcomes in hepatocellular carcinoma: an international cohort study
Source: Br J Cancer. 2020 Oct 19;124(2):407–13. doi: 10.1038/s41416-020-01116-9 (PMC7852559; doi:10.1038/s41416-020-01116-9)
Supplement: Supplementary file 1 — Supplementary Materials [file 41416_2020_1116_MOESM1_ESM.docx]

**Supplementary Table 1:** Effects of age and common prognostic factors on overall survival in complete-case analysis.

|  | **Univariable Models** | | | **Multivariable Models** | | | |
| --- | --- | --- | --- | --- | --- | --- | --- |
| **Predictor** | **Hazard Ratio (95% CI)** | ***P*** | ***N*** | **Hazard Ratio (95% CI)** | ***P*** | ***N*** | |
| **Full sample set of patients with HCC** | | | | | | |  |
| Age >75years | 1.00 (0.93 to 1.08) | 0.9689 | 5,598 | 0.99 (0.88 to 1.11) | 0.8352 | 2,754 | |
| BCLC stage C or D vs 0, A or B | 2.03 (1.86 to 2.21) | <0.0001 | 5,082 | 1.59 (1.39 to 1.81) | <0.0001 | 2,754 | |
| CTP class (B, C vs A) | 2.28 (2.14 to 2.44) | <0.0001 | 5,572 | 2.01 (1.82 to 2.22) | <0.0001 | 2,754 | |
| Tumour size >7cm | 1.87 (1.69 to 2.08) | <0.0001 | 3,383 | 1.63 (1.45 to 1.83) | <0.0001 | 2,754 | |
| PVT | 1.05 (0.98 to 1.12) | 0.1494 | 5,074 | 1.11 (1.02 to 1.21) | 0.0169 | 2,754 | |
| Presence of metastasis | 1.50 (1.37 to 1.64) | <0.0001 | 5,549 | 1.15 (1.00 to 1.33) | 0.0561 | 2,754 | |
| AFP > 400ng/dL | 1.93 (1.78 to 2.09) | <0.0001 | 5,266 | 1.69 (1.51 to 1.90) | <0.0001 | 2,754 | |
| HCV vs other aetiologies | 0.86 (0.82 to 0.91) | <0.0001 | 5,598 | 0.92 (0.85 to 1.00) | 0.0487 | 2,754 | |
| Starting dose (800 mg vs 200/400 mg) | 0.94 (0.89 to 1.00) | 0.0477 | 5,598 | 0.95 (0.87 to 1.03) | 0.1996 | 2,754 | |
| Continent (Asia vs USA/Europe) | 0.74 (0.64 to 0.85) | <0.0001 | 5,598 | 0.75 (0.64 to 0.87) | 0.0002 | 2,754 | |
| **Patients over 75years with HCC** | | | | | | | |
| BCLC stage C or D vs 0, A or B | 1.52 (1.29 to 1.78) | <0.0001 | 683 | 1.10 (0.84 to 1.43) | 0.5059 | 407 | |
| CTP class (B,C vs A) | 2.38 (1.94 to 2.93) | <0.0001 | 786 | 2.01 (1.47 to 2.73) | <0.0001 | 407 | |
| Tumour size >7cm | 1.64 (1.36 to 1.98) | <0.0001 | 528 | 1.56 (1.23 to 1.98) | 0.0003 | 407 | |
| PVT | 1.21 (1.00 to 1.45) | 0.0467 | 723 | 1.19 (0.92 to 1.52) | 0.1822 | 407 | |
| Presence of metastasis | 1.38 (1.16 to 1.64) | 0.0003 | 778 | 1.39 (1.04 to 1.85) | 0.0242 | 407 | |
| AFP > 400ng/dL | 1.77 (1.42 to 2.21) | <0.0001 | 705 | 1.48 (1.10 to 2.01) | 0.0105 | 407 | |
| HCV vs other aetiologies | 0.65 (0.54 to 0.79) | <0.0001 | 792 | 0.70 (0.53 to 0.93) | 0.0126 | 407 | |
| Starting dose (800 mg vs 200/400 mg) | 1.07 (0.92 to 1.25) | 0.3823 | 792 | 0.94 (0.75 to 1.19) | 0.6118 | 407 | |
| Continent (Asia vs USA/Europe) | 0.60 (0.45 to 0.79) | 0.0003 | 792 | 0.84 (0.61 to 1.16) | 0.2957 | 407 | |

Abbreviations: BCLC (Barcelona Cancer Liver Clinic), CTP (Child Pugh Score), PVT (portal vein thrombosis), AFP (α-fetoprotein), HCV (hepatitis C

**Supplementary Figure 1:** Kaplan-Meier curves illustrating the prognostic relationship of reason for sorafenib cessation.

**Supplementary Information 1:** Ethics committees in participating institutions that approved the study.

- Local Ethics Committee of the University Hospital of Freiburg, Germany.
- Institutional Review Board at the VA Connecticut Healthcare System (West Haven, CT) and the Corporal Michael J. Crescenz VA Medical Center (Philadelphia, PA).
- IRCCS Istituto Clinico Humanitas Local Ethical Committee, Milan, Italy
- Institutional Review Board of National Cancer Centre, Goyang, Korea
- Ethics Committee of the University Hospital Maggiore of Charity of Novara, Italy
- Kindai University Hospital Institutional Review Board, Japan
